# Current Applications of Artificial Intelligence in Neuro-Ophthalmic Imaging: A Narrative Approach

**DOI:** 10.3390/medsci14040422

**Published:** 2026-07-23

**Authors:** Raluca Eugenia Iorga, Vlad Constantin Donica, Ciprian Danielescu, Camelia Margareta Bogdănici, Răzvana Sorina Munteanu-Dănulescu, Andreea Dana Moraru

**Affiliations:** 1 Grigore T. Popa University of Medicine and Pharmacy, University Street No 16, 700115 Iasi, Romania; raluca-eugenia.iorga@umfiasi.ro (R.E.I.); ciprian.danielescu@umfiasi.ro (C.D.); camelia.bogdanici@umfiasi.ro (C.M.B.); andreea-dana.moraru@umfiasi.ro (A.D.M.); 2 Department of Gastroenterology, “L. Pasteur” Clinical Hospital, 28630 Le Coudray, France; razvanadanulescu@yahoo.com

**Keywords:** artificial intelligence, fundus photography, optical coherence tomography, optic nerve head, neuro-ophthalmology

## Abstract

**Background:** The eye provides a valuable window for the identification of neurological diseases, as pathological changes may involve both the retina and the optic nerve. Standard imaging of the retina and the optic nerve are non-invasive and are useful tools in investigating the integrity of the visual pathways. With population aging and the increasing burden of neuro-ophthalmic diseases, there is a growing need for tools that can assist clinicians in achieving rapid and reliable diagnoses. AI may facilitate the detection of specific neuro-ophthalmological conditions. **Methods:** This article presents a narrative review of previously published studies addressing the current applications of AI in selected neuro-ophthalmic conditions, with emphasis on their advantages, limitations, and challenges in screening, diagnosis, and disease progression assessment. **Results:** AI-based approaches provide valuable opportunities for the screening, characterization, and monitoring of optic nerve head abnormalities in neuro-ophthalmology. Recent advancements, particularly in deep learning and convolutional neural networks, have shown notable potential in interpreting fundus photography, optical coherence tomography and magnetic resonance imaging, supporting a comprehensive assessment of optic nerve structure and function. These systems are particularly promising because they can extract quantitative information from routine imaging modalities and may assist clinicians in detecting structural damage that is difficult to identify through conventional evaluation alone. **Conclusions:** AI-assisted ophthalmic imaging has shown promising diagnostic performance in identifying ONH abnormalities and retinal or optic nerve changes associated with neurological disease. At present, however, most applications remain within research, retrospective validation, or proof-of-concept settings, and evidence that AI consistently improves early diagnosis or clinical outcomes beyond conventional assessment remains limited. Accordingly, AI should currently be regarded as a complementary tool that may support screening, image interpretation, disease characterization, and referral decisions rather than replace established clinical evaluation.

## 1. Introduction

The eye provides a valuable window for the identification of neurological diseases, as pathological changes may involve both the retina and the optic nerve. Disorders affecting the visual pathways and ocular structures can initially manifest through symptoms such as optic neuritis or visual field abnormalities, which may be overlooked or incorrectly attributed to other causes. Timely recognition is essential for limiting disease progression and improving clinical outcomes. Therefore, the identification of robust biomarkers for early diagnosis remains a major priority, particularly because many neurological disorders are confirmed only after significant visual or neurological impairment has already developed [1,2].

Optic nerve abnormalities are frequently encountered in ophthalmic practice and represent an important reason for specialist evaluation. Accurate diagnosis and longitudinal assessment of disease progression require careful examination of the optic disc, a process that depends on the clinical expertise and interpretive skills of the ophthalmologist. Neuro-ophthalmology focuses on disorders affecting the visual pathway, which may be compromised by a broad spectrum of pathological mechanisms, including autoimmune, infectious, inflammatory, ischemic, and compressive processes. In some cases, an apparently isolated neuro-ophthalmic manifestation may be the first indication of an underlying neurological disorder. Moreover, optic nerve head swelling may represent the sole clinical sign of raised intracranial pressure, including in potentially life-threatening neurological conditions [3].

Standard imaging of the retina and the optic nerve (retinal fundus photography and optical coherence tomography [OCT]) are non-invasive and are useful tools in investigating the integrity of the visual pathways [4].

Advances in computational processing, mathematical modeling, and the availability of large-scale datasets have enabled artificial intelligence (AI), particularly through machine learning (ML) and deep learning (DL), to achieve increasingly strong performance in medical applications [5,6]. In ophthalmology, the routine generation of extensive clinical data from investigations such as fundus photography, optical coherence tomography (OCT), and automated perimetry provides an important foundation for AI-based analysis. By processing large volumes of complex information, AI may support clinicians in diagnostic interpretation and decision-making [7].

Retinal imaging and OCT complement conventional clinical ophthalmoscopy and contribute to the identification of neurological disorders involving the optic nerve. However, accurate interpretation of these imaging modalities requires substantial clinical expertise [8]. DL approaches have shown promising results in the automated assessment of retinal images, including the detection of papilledema associated with raised intracranial pressure [9]. Compared with subjective clinical evaluation and traditional diagnostic approaches, DL algorithms may allow faster and more accurate recognition of optic nerve disorders [10].

With population aging and the increasing burden of neuro-ophthalmic diseases, there is a growing need for tools that can assist clinicians in achieving rapid and reliable diagnoses [11]. Neuro-ophthalmology is challenging for AI because it needs a multidisciplinary approach. While AI excels in straightforward screening tasks, neuro-ophthalmic conditions are characterized by subtle visual manifestations and multimodal diagnostic requirements. AI-based systems have demonstrated high accuracy in the interpretation of fundus photographs, OCT scans, and magnetic resonance imaging (MRI). Consequently, AI may facilitate the detection of specific neuro-ophthalmological conditions, including optic neuritis, ischemic optic neuropathy, papilledema, and glaucomatous optic nerve damage.

While AI analysis may have an increased potential, clinical implementation faces significant barriers. Compared with retinal diseases such as diabetic retinopathy and glaucoma, neuro-ophthalmic disorders present greater challenges for AI development because they are often rarer, more heterogeneous, and associated with overlapping clinical and imaging findings. Their diagnosis frequently requires the integration of ophthalmic imaging with visual field testing, electrophysiology, neuroimaging, neurological examination, and systemic clinical data rather than relying on a single standardized image-based endpoint. These factors limit dataset size, complicate diagnostic labeling, and make external validation and clinical implementation more difficult. One of the main limitations is the quality diversity, and representativeness of training datasets, along with the validity and standardization of portable imaging devices [12].

Despite its considerable potential, the application of AI in neuro-ophthalmology remains subject to important limitations. Many available models have been developed using retrospective, single-center, or relatively homogeneous datasets, which may restrict their generalizability across populations, imaging devices, and clinical settings. Dataset imbalance and selection bias may further affect model performance, while limited explainability can reduce clinician confidence in algorithm-generated outputs. In addition, the lack of independent external validation, standardized evaluation protocols, and prospective clinical testing remains a major barrier to implementation. Regulatory requirements, data privacy concerns, interoperability, and the need for continuous post-deployment monitoring must also be addressed before these systems can be safely and reliably incorporated into routine clinical practice.

Overall, AI has the potential to support diagnostic practice in neuro-ophthalmology by facilitating earlier detection, improving diagnostic accuracy, and increasing the efficiency of visual pathway assessment. However, its clinical impact remains dependent on robust external validation, representative datasets, transparent model performance, and the resolution of regulatory and implementation challenges.

This review provides an integrated overview of the evolving role of AI in neuro-ophthalmology by bringing together areas that are frequently examined in isolation. It focuses on neuro-ophthalmic disorders and on retinal or optic nerve alterations that may serve as manifestations or biomarkers of neurological and neurodegenerative disease, rather than on primary retinal disorders. By examining AI applications across ophthalmology, neurology, and internal medicine within a unified analytical framework, the review identifies shared methodological and clinical challenges that may be less apparent in disease-specific or modality-specific analyses. It also summarizes the current state of evidence, highlights areas that remain insufficiently explored, and supports closer integration between neurological and ophthalmological practice.

The multidisciplinary nature of neuro-ophthalmology presents particular challenges for the development and implementation of AI-based systems. Accordingly, this review evaluates the main clinical tools and imaging modalities through which AI may support screening, diagnosis, disease characterization, and progression assessment. Unlike previous reviews that often focus on a single disorder, imaging technique, or AI application, the present synthesis examines these technologies across several neuro-ophthalmic conditions and neurological diseases with ocular manifestations. This cross-domain approach enables the identification of recurring limitations, including restricted external validation, limited comparisons with conventional assessment, dataset heterogeneity, and uncertainty regarding the incremental clinical value of AI.

## 2. Materials and Methods

**Figure 1:**
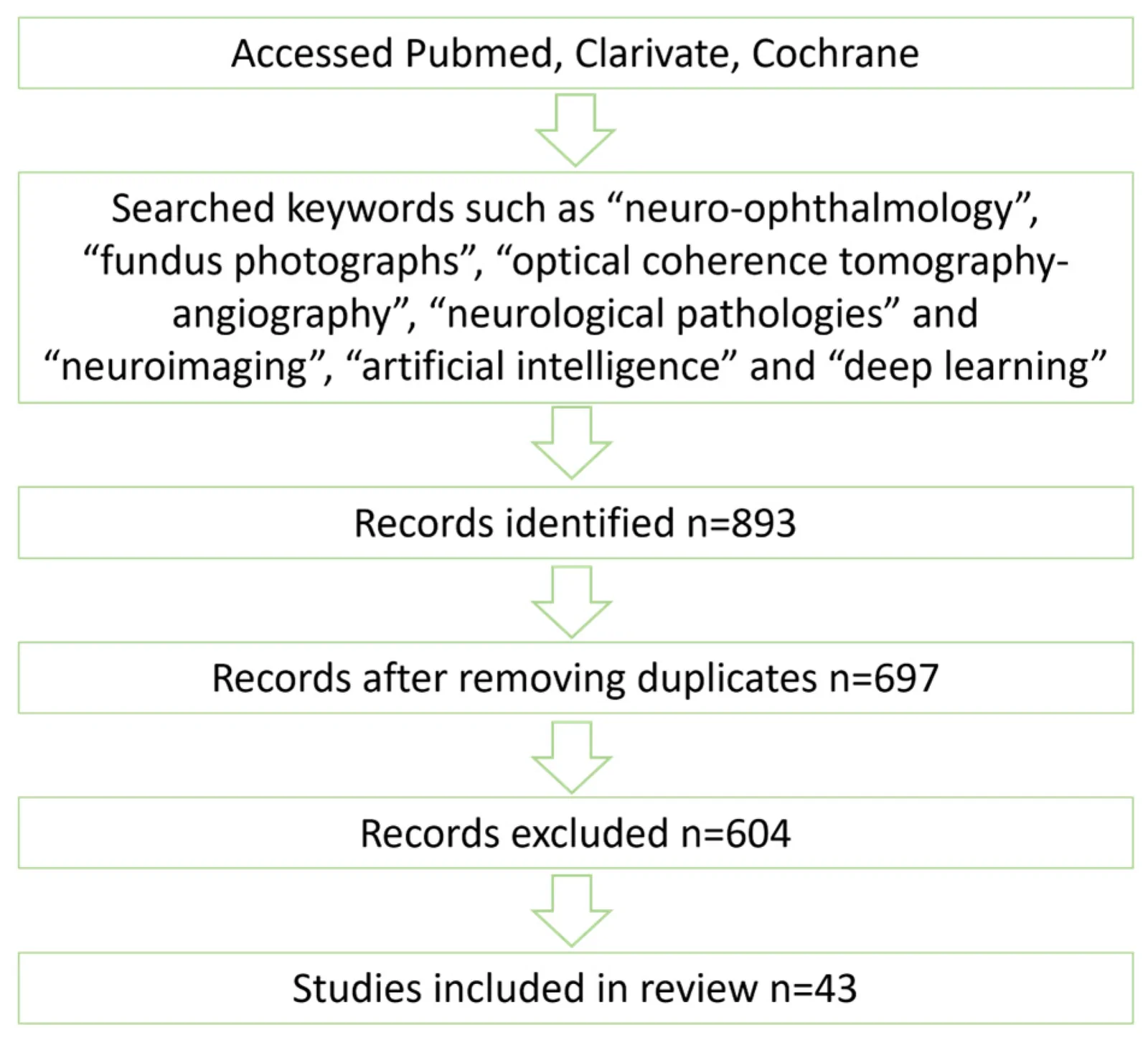
Flowchart.

A literature search was conducted using MEDLINE (PubMed), Web of Science (Clarivate Analytics), and the Cochrane Library (Cochrane) (Figure 1). We conducted the literature search by accessing PubMed virtually from 28 February 2026 to 29 April 2026. The search primarily targeted studies published within the last 10 years. Medical keywords such as “neuro-ophthalmology”, “fundus photographs”, “optical coherence tomography–angiography”, “neurological pathologies”, and “neuroimaging”, “papilledema” were combined with machine learning-related terms, including “artificial intelligence” and “deep learning”.

We included articles published after 2015. The inclusion criteria were the scientific quality of the publications, their relevance to neuro-ophthalmology, and their focus on fundus photos, OCT interpretation, and artificial intelligence. The main inclusion criteria were relevance to the topic of recent applications of AI in neuro-ophthalmology and analysis of the utility and diagnostic accuracy of AI in eye pathology. Studies were required to report sufficient data for diagnostic accuracy, sensitivity, specificity, and the area under the receiver operating characteristic curve (AUC). We included both retrospective and prospective studies. Preference was given to peer-reviewed studies with clear clinical applicability and reported diagnostic performance.

We excluded case reports, narrative reviews, editorials, conference abstracts without full-text publications. Duplicate records were removed, and articles with limited relevance were excluded after title screening. Two reviewers (REI and VCD) independently screened the titles and abstracts of all retrieved articles. The full texts of potentially eligible articles were assessed according to the inclusion criteria. Any disagreements regarding study inclusion were resolved by consensus or, if necessary, by a third senior reviewer.

Generative artificial intelligence tools were used solely to rework phrasing, improve grammar, and enhance the clarity, readability, and overall communication of the manuscript. The scientific content, interpretation of results, conclusions, and final responsibility for the article remain entirely with the authors. No generative AI tool was used to generate original research data, perform analyses, or replace the authors’ scientific judgment.

## 3. Machine Learning Versus Deep Learning

**Figure 2:**
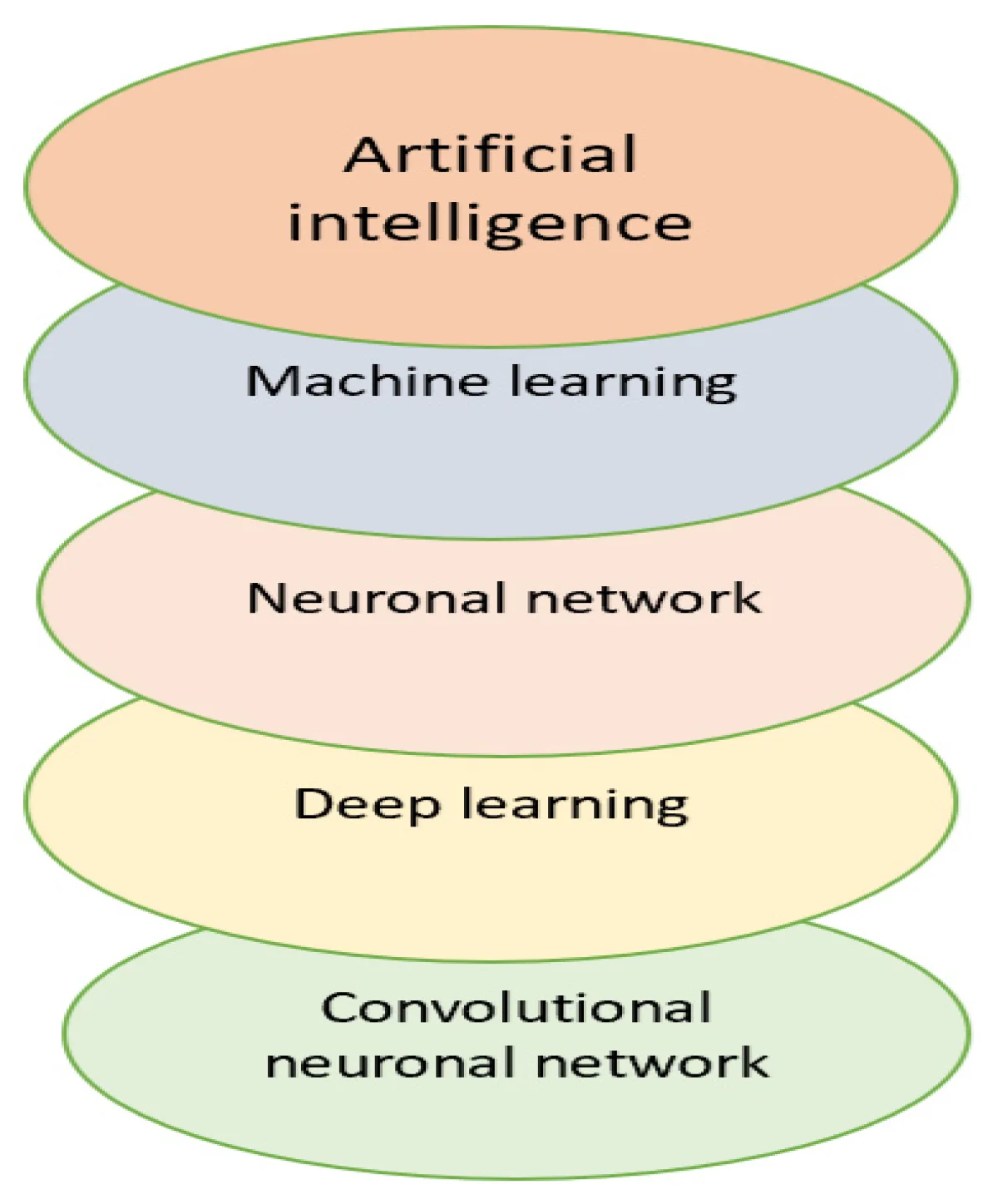
Stratification of each subcategory of AI.

The term AI was used in 1955 by John McCarthy to describe computer systems capable of performing complex tasks that only humans can do [13] (Figure 2). AI uses computer algorithms to learn from raw data and to create a representation of this data [14].

ML represents one of the main methodological foundations of AI, enabling computational systems to improve their performance by learning directly from data rather than relying exclusively on predefined instructions [15]. By analyzing datasets, ML algorithms can extract relevant patterns, generate decision rules, and apply these rules to new, unseen samples [16]. Neural networks are an important subgroup of ML models, built from interconnected computational units that are trained on large datasets to recognize features associated with specific outcomes [17]. As the number and complexity of these interconnected layers increase, these models evolve toward DL architectures, which are able to process more abstract and complex data representations [18,19] (Figure 2).

Traditional ML approaches have shown useful performance in solving classification and prediction tasks. However, their effectiveness may be limited when expert-level interpretation, contextual reasoning, or complex pattern recognition is required [20]. DL offers a more advanced approach by using multi-layered artificial neural networks capable of automatically identifying complex features within large datasets [21]. This reduces the dependence on manually defined features and extensive domain-specific input, although it requires substantial amounts of data and adequate computational resources [22]. In several medical applications, DL segmentation (DLS) have achieved performance comparable to that of human clinicians [23]. In medical imaging, convolutional neural networks (CNNs) are particularly well suited for analyzing spatial information, making them highly relevant for the interpretation of fundus photographs and OCT scans [24].

For neuro-ophthalmic image analysis, adequate preprocessing is essential before images are introduced into DL models. Common steps include resizing images to ensure uniform input dimensions, applying data augmentation to improve model robustness against variations in image acquisition, and normalizing pixel values to reduce variability across datasets [25]. These procedures contribute to more stable model training and may improve the reliability of DL-based predictions using imaging data.

### 3.1. Deep Learning Architectures

DL models rely on layered neural network architectures that progressively extract features from input data and refine their predictions through repeated exposure to labeled examples [15]. In ophthalmic imaging, CNNs are especially relevant because they can detect subtle structural patterns within the retina and optic nerve that may be difficult to recognize during routine clinical assessment. Architectures such as U-Net are commonly applied for segmentation tasks, whereas ResNet-based models are frequently used to improve image classification performance [26].

### 3.2. Deep Learning Model Development and Validation

The development of DL models requires careful organization of the dataset into training, validation, and testing subsets. When only limited data are available, cross validation during training may be used to improve reliability and reduce the risk of overfitting [27]. High-quality labeling is essential, particularly for diagnostic applications, because the reference standard determines how model predictions are evaluated [28]. Depending on the clinical objective, DL models may be designed to classify disease presence, estimate disease severity, or predict continuous outcomes through regression-based approaches.

### 3.3. Model Performance Evaluation

Diagnostic accuracy metrics are statistical measures used to evaluate how reliably a test distinguishes between the presence or absence of a disease or condition. In neuro-ophthalmology, heatmaps may enhance model interpretability by highlighting the image regions that contributed most strongly to the algorithm’s prediction. This visual feedback can help clinicians determine whether the model is focusing on clinically meaningful ocular features rather than irrelevant image characteristics [29].

### 3.4. Strengths and Pitfalls of Deep Learning Models

DL models are increasingly being explored as supportive tools in neuro-ophthalmology, particularly in settings where rapid image-based assessment may influence patient triage. For example, AI-assisted analysis of nonmydriatic fundus photographs has shown potential for identifying optic nerve abnormalities in acute care environments, allowing earlier recognition of patients who may require urgent specialist evaluation [30].

Despite these advantages, the clinical use of AI requires careful consideration of model-related limitations. The performance of these systems depends strongly on the quality, representativeness, and consistency of the data used during development. As in clinical research, bias may arise from measurement variability, confounding factors, or unbalanced training and validation cohorts [31]. Therefore, appropriate dataset selection and clearly defined output measures are essential to improve reliability and reduce systematic errors. In retinal image analysis, additional challenges may occur because CNN-based models can become overly dependent on image texture or minor visual details, increasing the risk of overfitting [32].

Incomplete or biased datasets may lead to models that reproduce or even amplify existing inequalities, especially when certain patient groups are underrepresented. Other concerns include limited model transparency, difficulties in clinical interpretability, ethical considerations, and the environmental impact associated with large-scale computational processing [33]. Methods such as cross-validation and stratified sampling may help reduce some of these limitations, but they do not eliminate the need for external validation and clinical oversight. Accordingly, AI should be regarded as an adjunct to, rather than a replacement for, specialist judgment in neuro-ophthalmic practice.

### 3.5. Multimodal Large Language Models

Multimodal Large Language Models (MLLMs) are revolutionizing neuro-ophthalmology by bridging the gap between clinical history and complex, multi-modality imaging (OCT, visual fields, and MRI). They generate differential diagnoses and streamlining the analysis of retinal structures that mirror central nervous system health. Ophthalmology offers a vast amount of datasets, like fundus photography, OCT, visual field, MRI, ideal for AI development [34].

These models, such as RETFound, RetiZero, EyeFM, and multimodal agents like ChatGPT-5 for ophthalmology, are capable of generalizing multiple tasks [35]. Foundation models are designed to perform multiple tasks, from disease detection to prognostic prediction, and even natural language report generation [36].

An AI agent can analyze an OCT scan, cross-reference it with the patient’s history, and generate a personalized treatment recommendation. Ha et al. investigated the capabilities of LLM for providing information and diagnoses in neuro-ophthalmology by comparing the performances of ChatGPT-3.5 and -4.0, Bard, and Bing. ChatGPT-3.5 and -4.0 are better than Bard and Bing in terms of answer success rate, answer quality, and critical keywords for diagnosis in ophthalmology [37].

Tao et al. evaluated the potential of GPT-3.5 to automate patient education handouts on common neuro-ophthalmic diseases. A trained neuro-ophthalmologist assessed 51 generated handouts across 17 conditions using the “Quality of Generated Language Outputs for Patients” (QGLOP) tool. The mean QGLOP score was 11.9 out of 16 points, suggesting a moderate level of satisfaction [38]. The use of LLMs in patient communication must be carefully monitored. Chen et al. found that physician responses often changed when using LLMs to answer patient questions, indicating automation bias [39].

### 3.6. Explainable AI (XAI)

Explainable AI (XAI) in neuro-ophthalmology translates opaque, “black-box” machine learning algorithms into transparent diagnostic tools. By using saliency maps and feature-guided prompting, XAI allows clinicians to see why an AI model flags a condition, such as optic neuritis or papilledema, on OCTs or fundus photos. XAI in papilledema focuses on creating interpretable DL algorithms that can detect, grade, and differentiate optic disc swelling. aiming to make the diagnostic process transparent, trustworthy, and actionable for clinicians. The research of Abbas et al. proposed an efficient transfer learning-based XAI model that enables transparent insights into ocular disease prediction. Their model achieved an accuracy rate of 95.74%, indicating its efficiency in enhancing diagnostic accuracy [40]. XAI utilizes post-hoc explanation methods like Grad-CAM (Gradient-weighted Class Activation Mapping) in order to visualize the exact regions of the optic nerve. XAI can correlate its predictions with standardized clinical benchmarks, such as the Frisén scale. By mapping heatmaps to specific Frisén stages, doctors can immediately verify if the AI’s grading aligns with their own clinical assessment [41]. Modern XAI frameworks like Eye-XAI integrate symptom-model correlation to clarify how demographic data, underlying symptoms, and image findings combine [42].

## 4. Application of AI in Optic Neuropathy of Fundus Images

The diagnosis of optic neuropathy is based on clinical signs and ancillary tests, such as colour vision evaluation, visual fields, optic nerve parameters on OCT and sometimes electrophysiology. An optic neuropathy can be associated with a normal appearance of the optic nerve head (ONH), or can be associated with swelling of the optic disc. With time, swelling of the optic disc can regress, or can evolve into atrophy.

Fundus images are widely used in clinical neuro-ophthalmic practice. Visualization of the optic nerve head ONH appearance with the help of direct ophthalmoscopy offers an important tool in the evaluation of a patient’s ocular and neurological status. Interpretation of these photographs is limited by human analysis and are mainly descriptive. AI could enhance disease classification and identify relevant features that were undetectable by expert neuro-ophthalmologists. Various methods exist for AI partitioning of fundus photos based on color, textural, vascular, and disc margin obscuration properties [43]. DL models have been trained to detect and grade papilledema using fundus images and have shown comparable performance to human interpretation [9]. The appearance of ONH depends on its structural integrity. Axonal loss typically causes ONH pallor, atrophy, cupping in glaucoma and swelling in ischemic, inflammatory, infiltrative, compressive neuropathies [21]. AI might represent a valuable and alternative tool to trained neuro-ophthalmologists, in order to offer a fast and accurate interpretation of ONH appearance [44]. The algorithm and performance of studies using AI to evaluate fundus photographs are presented in Table 1.

Liu et al. presented their research on high performance DLS using small data sets. The system modified the ResNet-152 deep CNN that was pretrained on ImageNet, which was able to distinguish between normal and abnormal optic discs and detect the optic disc laterality. This system detected various neuro-ophthalmic diseases such as atrophy, hypoplasia, optic papilledema and ischemic neuropathy [55]. Biousse et al. compared the DLS and neuro-ophthalmologists in the diagnosis of optic disc abnormalities. DLS performs at least as well as two professional neuro-ophthalmologists in the classification of optic disc appearance [52]. Liu et al. showed that a DL algorithm, when trained, can successfully distinguish optic neuritis from non-arteritic anterior ischemic optic neuropathy (NAION) [56]. Several studies have shown that DLS can accurately recognize the right or left eye in photos with optic discs [49,55].

### 4.1. Papilledema and Intracranial Pressure

Papilledema is defined as ONH swelling due to raised intracranial pressure. Papilledema is not necessarily associated with visual dysfunction and patients may present with headache or other signs characteristic for raised intracranial pressure. Papilledema is identified by fundus examination, by direct or indirect ophthalmoscopy. Pseudo-papilledema, including optic disc drusen and myelinated nerve fibers can mimic papilledema. DLS could improve the detection of papilledema.

A notable example in this field is the BONSAI-DLS, an AI-based DL system developed for optic disc assessment. Its architecture combines a U-Net segmentation component with a DenseNet classification network, allowing both localization and categorization of the optic disc [43]. The system was designed to differentiate normal optic discs from papilledema and other optic disc abnormalities [50]. In related image-analysis applications, U-Net-based CNN approaches have also been used for blood vessel extraction from fundus photographs [57].

In 2020, the BONSAI-DLS investigators trained their model using 14,341 fundus photographs collected from 19 different sites and evaluated its performance externally on 1505 additional images. The aim was to classify optic disc appearance as normal, papilledema, or other abnormality [50]. In the external-testing cohort, the model achieved an AUC of 0.96 for papilledema detection, with a sensitivity of 96.4% and a specificity of 84.7%.

An important issue is whether DLS-based assessment can match or exceed expert clinical interpretation. In a subsequent study, the performance of a ML algorithm was compared with that of two neuro-ophthalmology specialists using 800 newly selected fundus photographs [52]. The DLS showed comparable or superior accuracy, sensitivity, and specificity relative to the experts. BONSAI-DLS has also been applied successfully to nonmydriatic fundus photographs obtained in emergency room settings, although images acquired after mydriasis appeared to support more accurate recognition.

Nevertheless, much of the available evidence is derived from retrospective datasets used for model training and validation [25]. This limits the ability to fully assess real-world performance. Therefore, large prospective studies are needed to evaluate these algorithms under routine clinical conditions [58]. More recent studies using smaller testing datasets have further supported the accuracy of trained DLS for detecting papilledema and other ONH abnormalities [53,57].

Vasseneix et al. developed a DLS for automated papilledema severity assessment using 2103 mydriatic fundus photographs from patients with increased intracranial pressure. Papilledema was grouped according to Frisen grading, with grades 1–3 considered mild to moderate and grades 4–5 classified as severe [9,59]. Distinguishing between severity categories is clinically relevant, as it may help estimate visual prognosis and assess response to treatment over time [60,61]. The results were comparable to that of neuro-ophthalmology experts [9].

### 4.2. Optic Nerve Head Pallor

The ONH can look pale or atrophic in patients with chronic optic neuropathies [62]. Given their universal availability fundus photography may be used as an initial method of screening for optic nerve atrophy, examiners being able to distinguish between normal, moderate and severe optic atrophy [63]. Yang et al. used brightness correction ratio and background region as parameters for a computer-aided detection system to detect ONH pallor using colour fundus images achieving high sensitivity, accuracy and specificity [48].

### 4.3. Glaucomatous Versus Non-Glaucomatous Optic Neuropathy

To differentiate between glaucomatous optic neuropathy (GON) and non-glaucomatous optic neuropathy (NGON) due to compression, hereditary diseases, chronic ischemia, inflammation, trauma, or toxic, Yang et al. utilized a DL algorithm. Their study analyzed over 3.000 retinographies using a CNN of the ResNet-50 architecture achieving an overall accuracy of 99.1% for detecting healthy controls, GON and NGON [51]. This performance is better compared to a study where neuro-ophthalmologists and glaucoma specialists identified correctly from fundus photos only 75% of glaucoma cases [64].

### 4.4. Optic Disc and Systemic Diseases

Information extracted from retinal fundus images can be used to predict cardiovascular risk factors, since retinal vascular parameters reflect systemic microcirculatory health. Retinal vessel diameters and flicker light-induced dilation are emerging as potential biomarkers for cardiovascular risk prediction, while retinal microvascular changes may appear before clinically evident disease. Beyond cardiovascular disorders, retinal vessel analysis also shows promise in degenerative cerebral diseases, renal dysfunction, pregnancy-related conditions, and sleep apnea. Its greatest clinical value may come from automated AI-based analysis integrated into broader risk assessment tools, using the predictive value and wide availability of retinal photography, including smartphone-based platforms [65,66]. A DL model predicted with an excellent accuracy the cardiovascular risk factors using features such as the optic disc and retinal vascularization [67]. Poplin et al. trained data from 284,335 patients and validated the DL model on 2 independent data sets of 12,026 and 999 patients. This model used the anatomical characteristics of the optic disc to predict cardiovascular risk factors, such as age, gender, smoking status, systolic blood pressure, and major adverse cardiac events [67]. This is very important, as these risk factors are not discernible by clinicians from fundus ophthalmoscopy.

For a long time, the retina has been considered a potential candidate for detecting neurodegenerative conditions, as it is affected by ageing and neurodegenerative processes [68]. The ageing retina is characterized by neuronal loss which causes retinal thinning, and vascular dysfunction. These changes can be quantified with the help of OCT and OCTA [69,70]. OCTA may help find vascular changes in Alzheimer disease (AD), representing an indirect biomarker, the variability of its parameters is large and its disease specificity is not yet known [71].

Zhang et al. studied whether ML could discriminate patients with dementia from normal individuals on standard retinal fundus photographs [72]. Their model achieved a high capacity of classifying patients with dementia and normal individuals. Tian et al. developed a classification model using CNN, based on retinal photographs, particularly on the architecture of retinal vessels [73]. The model was able to discriminate AD dementia patients from normal controls with increased sensibility and specificity. A recent study aiming to test a retinal photograph-based DL algorithm for AD dementia identification, included a total of 12,949 retinal photographs from 648 AD dementia patients and 3240 controls with no dementia [74]. The performance of the DLS in 5 independent external cohorts achieved accuracies ranging from 79.6% to 92.1%, and AUROCs ranging from 0.73 to 0.91.

The fact that DLS performed well in the presence of concomitant eye diseases, may suggest that AD dementia might be associated with specific retinal features. AI-based retinal imaging may represent an interesting objective tool in screening elderly patients, in combination with other validated modalities.

## 5. Application of AI in Optic Neuropathy of OCT Images

OCT imaging of the retina represent a low-cost and accessible method for identifying early neurodegenerative changes [75,76]. ML algorithms trained on OCT images can detect early changes in retinal morphology that correlate with neurological pathology [77]. OCT is an important tool for diagnosis, disease progression monitoring, treatment efficacy, and it helps in determining retinal layer thinning or optic disc swelling in specific neuro-ophthalmic pathologies. OCT generates high-resolution cross-sectional images of internal structures, measuring light amplitude, providing detailed imaging of retinal layers [78]. The multimodal use of OCT with fundus photography and other modalities such as MRI and VEP may provide additional data regarding the integrity of the optic path in neuro-ophthalmological conditions [63,79,80]. To enable ML algorithms to detect and classify conditions affecting specific retinal layers, these must first be delineated. Study objectives, algorithm and performance are presented in Table 2.

### 5.1. Positioning of the Optic Disc

Thompson et al. used the spectral-domain optical coherence tomography (SD-OCT) Bruch’s membrane opening minimum rim width parameter as the reference standard for optical disc photo marking [88]. This has limitations, for example in the case of optic disc swelling, where there are shadow artifacts, segmentation of projected retinal vessels from the volume of the OCT may be challenging. Islam et al. suggested that vascular visibility can be improved by vascular information from multiple projected retinal layers. They designed a method based on DL to segment vessels, which involves simultaneously using three front images of the OCT as input. This way, they achieved better vascular segmentation by using multiple frontal images [89].

### 5.2. Cup Disc Ratio

Optic disc measurements obtained with Cirrus HD-OCT may provide useful input data for training CNN-based models, particularly when incorporating CDR values. Mwanza et al. showed that CDR assessment using HD-OCT had strong diagnostic performance for glaucoma, comparable to average and clock-hour RNFL thickness measurements [90]. Other studies comparing Cirrus HD-OCT with confocal laser ophthalmoscopy have reported a strong correlation between the two imaging approaches [91,92]. Although OCT-derived parameters may still be affected by measurement errors, Cirrus HD-OCT offers reliable and reproducible CDR data and may provide greater precision than clinician-estimated VCDR [93,94].

### 5.3. Retinal Nerve Fiber Layer

SD-OCT is widely being used as a tool to identify optic nerve structural damage. Optic disc and RNFL measurement are commonly used in neuro-ophthalmology to monitor disease diagnosis and progression [95]. SD-OCT structural damage assessment requires segmentation of the region of interest to extract measurements, such as the RNFL thickness. This measurements are done by the OCT software, but might present segmentation errors and artifacts in the RNFL scanning [96]. Another challenge for ophthalmologists is the interpretation of the SD-OCT scanning, which requires the analysis of many parameters and different regions.

Given the limitations associated with OCT segmentation, DL approaches may offer an alternative method for quantifying structural damage directly from imaging data. Rather than depending on predefined parameters generated by automated segmentation software, these models can analyze original SD-OCT scans and extract clinically relevant information from the image itself. This may be particularly useful in cases where conventional segmentation fails, as DL models have been shown to recover reliable RNFL thickness information from such images [97]. Petersen et al. similarly reported that a DL model using non-segmented SD-OCT data outperformed automatically segmented RNFL thickness parameters in glaucoma detection [98].

Jammal et al. developed a machine-to-machine (M2M) DL algorithm trained on SD-OCT-derived RNFL thickness parameters and applied it to 490 fundus photographs from 370 subjects. The predicted structural information was correlated with the global index from SAP, and the algorithm showed superior performance to human assessment in identifying eyes with repeatable glaucomatous visual field loss [99]. Medeiros et al. further demonstrated that quantitative SD-OCT data can be used to train a DL CNN to estimate glaucoma-related structural damage from optic disc photographs. Their model predicted average RNFL thickness from optic disc images, showing a strong correlation with SD-OCT measurements, while achieving AUC values comparable to those based on measured RNFL thickness [100].

### 5.4. Stereoscopic Optic Disc Image

Using the original and undivided OCT volume of the optic disc, Maetschke and colleagues developed a DL algorithm which can distinguish glaucoma eyes from healthy eyes. The AUC was 0.94, while the AUC of a logistic regression model combined with SD-OCT parameters was 0.89 [101].

The optic disc pit (ODP) is a rare ophthalmic finding. Maertz et al. proposed a new OCT technique that can be used to display the optic papilla imaging of ODP using A-scans at 1.68 million per second. 3D volume reconstruction of the ODP can be used to generate a 3D rendering of the optic disc region, thus helping the study ODP characteristics [102]. Although DL algorithms may capture information that might be undetected by the human eye, it is necessary to be aware of the resolution limitations.

### 5.5. Applications of AI in Specific Neuro-Ophthalmic Conditions

#### 5.5.1. Optic Neuritis in Multiple Sclerosis

Multiple sclerosis (MS) is a complex autoimmune disorder characterized by central nervous system (CNS) demyelination. OCT in MS is characterized by thinning of the axonal and neuronal retinal layers. The differentiation of MS from other CNS demyelinating disorders might be challenging. DL models could distinguish between disease types using MRI [103]. Ciftci Kavaklioglu et al. support the use of ML on OCT to differentiate MS in children from other demyelinating disorders [82].

OCT-based ML models have also been investigated as diagnostic support tools in MS. In one study, a support vector machine approach combining OCT parameters with low-contrast visual acuity showed strong performance in distinguishing patients with MS from healthy controls [83]. Another study using a CNN model trained on raw peripapillary ring scan data demonstrated the feasibility of identifying previous optic neuritis in patients with MS and performed better than retinal thickness measurements alone [81]. AI can potentially be applied to aid in the diagnosis of hard-to-detect conditions, like sub-clinical optic neuritis in MS. This is particularly useful if AI is paired with traditional OCT biomarkers of MS [104,105]. A recent study suggested that a 5-µm inter-eye difference in peripapillary RNFL thickness and a 4-µm inter-eye difference in GC-IPL thickness are sufficient to diagnose a history of unilateral optic neuritis in MS patients [83]. Another program was accurate in diagnosing pediatric CNS demyelinating pathologies, using the OCT measurements of GC-IPL thickness in the supertemporal sector [82].

#### 5.5.2. Optic Neuritis and Other Neuropathies

AI-driven OCT evaluation could aid in differentiating between different causes of optic neuropathy as well as demyelinating optic neuritis. ML classifiers have demonstrated excellent potential for distinguishing between NAION and optic neuritis (ON) in MS in the acute phase [87].

AI models have also demonstrated improved performance in distinguishing glaucomatous eyes and glaucoma severity using OCT-angiography images [84,86,106]. Another DL model using OCT images of the ONH classified with excellent performance papilledema, optic disc drusen, and healthy controls [85].

In another study, authors compared ischemic optic neuropathy with optic neuritis, using an AI model that measured vessel density using OCT-A. The researchers applied custom measurements of the peripapillary region of the optic disc and achieved high accuracy [87].

The use of OCT in clinical practice presents significant challenges and opportunities for further AI applications. Beyond disease detection and classification tasks, ML algorithms can identify and segment retinal layers on OCT, saving time for clinicians [107]. AI applications in OCT-based neuro-ophthalmology have shown remarkable promise in disease diagnosis, but it is important to integrating these technologies into clinical practice.

## 6. Applications of Radiomics and AI Modalities in Neuro-Ophthalmology

Recent advancements in neuroimaging have given the opportunity to identify structural optic path changes, long before they become clinically evident or can reveal early microstructural damage in both brain and optic nerve tissues that previously went undetected [108,109]. DL, including CNNs, are capable of performing tasks such as brain and optic nerve segmentation, volume measurement, and pathology identification with very good accuracy [110]. Thus, AI provides aid in the identification of early neurodegenerative and neuro-ophthalmic changes in asymptomatic individuals, facilitating diagnosis and treatment [111].

AI systems will help radiologists increase throughput and decrease volume burdens. The major automated domain in which radiological research has mainly focused on include “Radiomics” and “Machine Learning” [112].

Radiomics is an emerging field combining “radiology” and “-omics,” which describes the processing of high volume of data through high throughput analysis [112]. Radiomics transform morphological data into quantitative metrics, helping detect abnormalities that are often imperceptible to the human eye and enhances the accuracy of the diagnosis [113,114]. Radiomics focuses on the extraction of quantitative features from radiological images, lesion shape, texture, intensity, and spatial relationships. These features are then introduced into ML algorithms, which identify patterns, classify disease states, or predict clinical outcomes based on training data [115].

ML software is used in radiology to train in lesion detection and to evaluate other distinct radiographic findings. Every input helps to improve detection accuracy and speed for future imaging [116]. DL models proved successful at developing higher quality and clearer reconstructed images by reducing noise and removing artifacts [115,117].

Magnetic resonance imaging MRI has an important role in diagnosing these pathologies, as it can detect white matter abnormalities. Nevertheless, several conditions can mimic the radiological features of demyelination, making the diagnosis difficult. These conditions include vascular leukoencephalopathy, small vessel ischemic disease, central nervous system CNS infections, neoplasms, and metabolic disorders [118,119]. Accurate differentiation between demyelinating diseases and mimickers is crucial as treatment strategies vary widely.

### 6.1. Multiple Sclerosis MS

MS diagnosis is currently based on the McDonald Criteria, which integrate clinical and paraclinical evidence of lesion dissemination in space and time [120]. Given the recent introduction of the optic nerve as a location for dissemination of demyelinating lesions, an increasing interest for its study has been observed targeting OCT, MRI and VEP investigations [63,79,121].

#### 6.1.1. Radiomics in MS

Radiomics analysis of MRI data displayed predictive values of 80% when used to predict EDSS, offering more precise outcomes in patients with MS [122]. In 2021, using a ReliefF algorithm, Peng et al. managed to obtain a prediction for MS lesion evolution [123]. Radiomics showed potential in identifying MS patients with cognitive impairment based on cortical lesions [124,125]. Moreover, radiomic models identified successfully active plaques in T2 FLAIR imaging, assessing MS disease activity [126,127]. Another study on pediatric MS patients applied radiomics to T2 FLAIR imaging, demonstrating significant lesion detection [128].

#### 6.1.2. Machine Learning in MS

In 2020, an algorithm integrating multiple ML techniques was developed in order to predict 2-year clinical disability in MS patients. The model used FLAIR imaging sequences combined with patient demographic data to generate its prediction accuracy, achieving a mean EDSS score error of 1.7 [129]. Some studies used ML and DL algorithm to recognize specific patterns of pathological development that determine prognosis severity. Eshagi et al. and Pontillo et al. found that T2-hyperintense lesions primarily involved in deep gray matter could lead to worse prognosis [130,131]. DL reconstruction enhanced the visualization of MS lesions on brain MRI, helping detect a higher number of lesions, compared to conventional MRI [132]. Regarding the differential diagnosis using ML, similar to radiomic studies, the majority has focused on diagnosing MS and Neuromyelitis optica spectrum disorder (NMOSD) [133].

### 6.2. Neuromyelitis Optica Spectrum Disorder

NMOSD is an uncommon autoimmune inflammatory disorder strongly associated with antibodies targeting AQP4. The presence of these antibodies contributes to immune-mediated injury involving the optic nerves and spinal cord [134].

#### 6.2.1. Radiomics in NMOSD

In 2019, Ma et al. developed a nomogram incorporating radiomic features and routine MRI measurements in order to differentiate NMOSD from MS in clinical practice. Their nomogram achieved an AUC of 0.9880 and 0.9363 for the primary and validation cohorts, respectively [135]. Liu et al. also developed a nomogram in order to predict MS and NMOSD using features such as lesion length, sex, and EDSS, combined with clinical and MRI data. Their model showed strong differentiation between MS and NMOSD, achieving an AUC of 0.8808 and 0.7115 in the primary and validation cohorts [136]. In 2021, Yan et al. extracted radiomic features from quantitative susceptibility imaging QSM images of MS and NMOSD patients and combined them with ML models and demographic data showing that radiomics features alone displayed better discrimination performance than demographic features. When combined, the model achieved higher discrimination performance characterized by an AUC of 0.927 [137]. In 2024, extracted radiomic features were highlighted from the hippocampus of NMOSD, MS, and control patients and were used for comparison. Patients with NMOSD and MS exhibited distinct patterns in the hippocampus, an area not typically associated in the pathophysiology of the two conditions [138].

#### 6.2.2. ML in NMOSD

Some studies revealed that the application of CNNs has shown promise in identifying subtle differences in lesion distribution and morphology between NMOSD and MS, with very good accuracy, sensitivity, and specificity [103,139]. Different studies compared the accuracy of CNNs to physician interpretations. The results suggest that CNNs can outperform traditional diagnostic methods when analyzing brain MRI data, including FLAIR, T1, and T2-weighted images [140]. Spinal cord segmentation with DL methodology has shown promise as an alternative tool in differentiating MS from NMOSD [141,142].

### 6.3. MOG Antibody-Associated Disorder (MOGAD)

MOGAD is an immune-mediated inflammatory disorder of the CNS associated with antibodies directed against MOG. In contrast to MS and NMOSD, MOGAD is more frequently preceded by an infectious prodrome [143].

#### 6.3.1. Radiomics in MOGAD

Recent studies have explored the value of MRI-based radiomics for distinguishing MOGAD from other acute demyelinating syndromes, particularly in pediatric populations. In a 2024 study, lesion features extracted from multiple MRI sequences were integrated into a fusion model, which achieved an AUC of 0.867 during internal testing and 0.810 during external validation [144]. These findings suggest that radiomics may provide additional quantitative information for differentiating MOGAD from other neuro-inflammatory disorders.

Li et al. also developed multiparametric MRI radiomics models using T1WI, T2WI, FLAIR, and a combined model. Their radiomics-based approaches outperformed the clinical prediction model in the training set, reaching higher AUC values, including 0.905 compared with 0.289 for the clinical model [145].

#### 6.3.2. Machine Learning in MOGAD

Huang et al. investigated a DL approach using combined brain and spinal cord MRI sequences, including coronal T2-weighted and sagittal T2-FLAIR images, to account for differences in lesion morphology and anatomical distribution. The best results were obtained with a fusion model integrating both brain and spinal cord information, which reached an AUC of 0.803 and an accuracy of 81.4% for MOGAD classification [146]. These findings support the potential role of DL-assisted MRI analysis in improving the recognition of MOGAD among demyelinating disorders.

Overall, AI-assisted neuroimaging and radiomics expand the diagnostic and prognostic value of MRI in demyelinating disorders. Across MS, NMOSD, and MOGAD, these approaches support lesion detection and characterization, assessment of disease activity and disability, and differentiation between clinically overlapping conditions. By integrating quantitative imaging features from the brain, optic nerve, and spinal cord with clinical data, AI may contribute to more precise disease classification, individualized prognosis, and improved treatment planning.

## 7. AI-Assisted Imaging and Neurodegenerative Diseases

Because the retina shares important structural and physiological characteristics with the brain, it has been increasingly investigated as a potential site for detecting changes associated with neurodegenerative disorders. Neuronal loss and retinal thinning may occur during aging and neurodegeneration, and these changes can be quantified non-invasively using OCT. Therefore, the use of AI algorithms has been increasingly used to detect neurodegeneration in diseases such as AD and Parkinson disease (PD) [68].

### 7.1. Alzheimer Disease

Aβ protein deposits, phosphorylated tau proteins vascular, dysfunction, changes in collagen content and increased cellular apoptosis are also seen in AD. Zhang et al. studied whether ML could discriminate patients with dementia and AD, from normal individuals on standard retinal fundus photographs [72]. The best model achieved an AUROC of 0.86, sensitivity of 92.8% and specificity of 69.6% in classifying patients with dementia and normal individuals. Wisely et al. investigated a mix of multimodal retinal images and patient data to create a CNN to identify symptomatic AD [77]. On the validation set, the best-performing model, which included GCL maps, quantitative data and patient data—reached an AUROC of 0.861. AlzEye study includes more than 6 million retinal images collected from hundreds of thousands of patients, aiming to identify novel retinal signatures in patients with AD dementia and cardiovascular diseases [147]. A recent study aimed to test a retinal photograph-based DL algorithm for AD dementia identification, using multiethnic and multinational datasets (12,949 retinal photographs from 648 AD dementia patients and 3240 controls) [74]. The classification performance of the DLS in 5 independent external cohorts achieved accuracies ranging from 79.6% to 92.1%, and AUROCs ranging from 0.73 to 0.91.

### 7.2. Parkinson Disease

Longitudinal studies demonstrate that PD patients exhibit progressive retinal thinning, particularly in the RNFL and macular regions, as well as foveal avascular zone (FAZ) enlargement. These findings correlate with motor and cognitive decline [148,149]. OCT thus offers a critical opportunity to identify preclinical biomarkers. Most studies focused on isolated OCT biomarkers, neglecting the integration of clinical variables, which could enhance diagnostic specificity [150]. There was an urgent need for robust, multimodal frameworks that harmonize OCT data with clinical features. Ganji et al. developed an explainable AI framework integrating retinal OCT biomarkers and clinical data to diagnose Parkinson’s disease (PD) with 95.3% accuracy and 0.98 AUC. The authors used data from 1176 participants, the model identifying SUPERIOR4 thickness (<120 µm) and foveal volume expansion (>0.15 mm^3^) as key biomarkers [151]. In a prospective study, the authors applied a DL algorithm to analyze fundus photographs from PD and non-PD participants. This algorithm helped identify crucial clinical features for diagnosing PD, correlating motor symptoms with retinal abnormalities [152]. Richardson et al. applied CNNs to train, validate and test OCT retinal images from both PD patients and healthy controls. The best-performing model demonstrated that CNNs detected retinal changes associated with Parkinson’s disease with an AUC of 0.918, 100% sensitivity, and 85% specificity [153].

Overall, AI-assisted retinal imaging may have clinical value as a non-invasive screening tool for identifying individuals at increased risk of AD or PD, supporting earlier referral and further neurological assessment. It may also facilitate the detection of subtle retinal changes before advanced clinical manifestations develop and provide objective biomarkers for monitoring disease progression. However, its role in population screening, early diagnosis, and longitudinal follow-up must be confirmed through prospective studies demonstrating disease specificity and added value beyond conventional clinical assessment.

## 8. Limitations and Challenges of Current AI Applications in Neuro-Ophthalmology

AI applications in neuro-ophthalmology face some challenges. In a recent review, Oshika showed that AI is set to enhance diagnostic accuracy, optimize treatment strategies, improving patient care. The authors suggest that even if AI offers substantial benefits, challenges remain, including the need for high-quality images, accurate manual annotations, patient heterogeneity considerations, and the “black-box phenomenon” [154].

One of the main limitations is the quality, diversity, and representativeness of training datasets, along with the validity and standardization of portable imaging devices. AI bias may occur when training datasets are unbalanced (insufficient data for certain subpopulations). As a possible solution, such databases may be modified by adding clinician-annotated labels and constructing an artificial scenario of data imbalance. Rare optic neuropathies are frequently not well represented in large datasets, risking model performance and overfitting, where the model works well on the training data but does not generalize to new, diverse patient populations. Bias and fairness are also key concerns. Models learned from homogeneous or unrepresentative data could present performance differences in diagnostics across various demographic groups.

Another limitation is the variability between AI systems, imaging devices, acquisition protocols, and preprocessing methods. Algorithms developed for one technology may produce false-positive and false-negative rates that differ from those obtained with another platform, even when evaluating the same pathological change. Consequently, outputs generated by different systems may not be directly comparable or interchangeable, potentially affecting diagnostic classification and the detection of clinically significant progression. Standardized acquisition protocols, harmonized outcome definitions, and cross-platform validation are therefore required before AI-derived measurements can be reliably used across devices and clinical settings.

Although several studies have reported high AUC, sensitivity, and specificity values, these findings should be interpreted cautiously, particularly when they are derived from small, retrospective, single-center, or highly selected datasets. Such results may be influenced by overfitting and spectrum bias and may not be reproducible across different populations, imaging devices, or clinical settings. Therefore, a distinction should be made between proof-of-concept models evaluated through internal validation and algorithms supported by independent external, multicenter, or prospective validation. Moreover, clinical readiness cannot be determined solely from diagnostic accuracy, as successful implementation also requires adequate calibration, robustness, explainability, fairness, workflow compatibility, and evidence that the model improves clinical decision-making beyond conventional assessment. Main AI limitations are presented in Table 3.

### Ethical and Medico-Legal Challenges of AI Implementation

AI has several related ethical aspects that need to be identified, as AI technology may compromise patient preferences, safety, and privacy. AI and big data pose threats to the fundamental human right to privacy, as ML collects and saves data from multiple sources [155].

Data privacy, cybersecurity and data sharing are major concerns in the implementation of ML and DL algorithms. While, in ophthalmology there are large imaging datasets, clinical implementation of AI analysis is faced with major practical and regulatory barriers. In addition, approval pathways remain complex, especially if constant updates are required after implementation. Although the anonymized datasets have been a benefit for the future technology, they also represent a significant risk and can cause major harm. Even for datasets not involving medical images, it may be possible to identify individuals by linkage with other datasets [156].

The principle of non-maleficence refers to the fact that physicians must maintain a high quality of life for their patients by respecting their wishes. In terms of AI, the principle of autonomy mainly applies to “brain-computer interface” (BCI), which involve direct communication between the brain and various external devices. Thus, signals coming from the brain are converted into commands for these external devices. AI development and sharing procedures are linked to justice aspects. BCIs are now available to patients, thus it is mandatory to have the informed consent from the patient. He must have knowledge regarding the device design, use, and potentially conflicting requirements. The ethical concern in this case is that the perspective of disabled people is likely to be disregarded [157].

The “black box”, also known as opacity, is suggestively exemplified by deep neural networks. If input data are provided, a neural network trained on a large dataset can find a pattern in the input data and make an output decision. The problem is that it is unable to explain how it reached that result. The neural network modifies its decision-making process, when given supplementary data. This opacity can lead to possible legal problems [158].

Developing strong regulatory mechanisms and ethical protocols is necessary to assure the safe, responsible, and equitable use of AI in neuro-ophthalmology [159].

## 9. Perspectives and Future Directions

AI-based approaches provide valuable opportunities for the screening, characterization, and monitoring of ONH abnormalities in neuro-ophthalmology. By integrating fundus photography, OCT, and relevant clinical data, DL models may support a more comprehensive assessment of optic nerve structure and function. These systems are particularly promising because they can extract quantitative information from routine imaging modalities and may assist clinicians in detecting structural damage that is difficult to identify through conventional evaluation alone. An overview for each medical device available for AI analysis is presented in Table 4.

The future clinical value of AI may depend not only on its ability to recognize optic nerve abnormalities, but also on its capacity to suggest the most likely underlying pathology and estimate disease progression or treatment response. Multimodal models combining patient-specific clinical information with imaging-derived biomarkers may improve diagnostic accuracy and support individualized management. However, before these systems can be widely adopted, further prospective and multicentric studies are needed to confirm their reliability, generalizability, and real-world utility in neuro-ophthalmic practice.

Future studies should include direct, head-to-head comparisons between AI-based models and conventional clinical assessment to determine whether AI provides a measurable advantage in the early detection of neurodegenerative changes. Such investigations should compare AI performance with established approaches, including clinician interpretation, standard OCT parameters, fundus examination, visual field testing, and conventional neuroimaging, using predefined outcomes such as diagnostic accuracy, sensitivity, specificity, and AUC. Particular attention should be given to determining whether AI can identify disease-specific retinal or optic nerve patterns rather than nonspecific neuroretinal alterations associated with aging or other ocular disorders. Prospective, externally validated, and multimodal studies are therefore needed to establish whether AI offers incremental clinical value beyond currently available diagnostic methods.

## 10. Conclusions

AI-assisted ophthalmic imaging has shown promising diagnostic performance in identifying ONH abnormalities and retinal or optic nerve changes associated with neurological disease. At present, however, most applications remain within research, retrospective validation, or proof-of-concept settings, and evidence that AI consistently improves early diagnosis or clinical outcomes beyond conventional assessment remains limited. Accordingly, AI should currently be regarded as a complementary tool that may support screening, image interpretation, disease characterization, and referral decisions rather than replace established clinical evaluation.

The translation of DL systems into routine neuro-ophthalmic practice is constrained by limited external validation, dataset bias, reduced interpretability, regulatory and ethical concerns, and uncertainty regarding real-world clinical utility. Prospective, multicenter studies are needed to determine whether these systems remain accurate across different populations, imaging devices, and clinical settings and whether their use improves patient management. Future developments may include multimodal platforms integrating fundus photography, OCT, visual fields, neuroimaging, and clinical data, as well as generative AI applications for education and symptom visualization. However, these approaches remain largely prospective and require careful validation before clinical implementation.

## Figures and Tables

**Table 1:** Comparison of AI model and performance in neuro-ophthalmology studies using color fundus photographs.

Authors	Objective	Algoritm	Datasets	Performance Characteristics
Echegaray et al. (2011) [45]	To grade the severity of papilledema based on MFS and compared to trained neuroophthalmologist	Classical machine learning	294 images taken from patients with diagnosed papilledema	/
Agne et al. (2015) [46]	To predict ONH volume from fundus photographs	Classical machine learning	48 images showing optic nerve edema	/
Akbar et al. (2017) [47]	To detect papilledema from normal optic discs Grading papilledema severity into Mild (MFS 1 and 2) and Severe (MFS 3 to 5)	Classical machine learning	160 images including 50 normal and 40 with papilledema from the publicly available STARE database, and 40 normal and 30 with papilledema from local database.	Accuracy 92.9%/97.9%
Akbar et al. (2017) [47]	To detect papilledema from normal optic discs	Classical machine learning	160 images including 50 normal and 40 with papilledema from the publicly available STARE database, and 40 normal and 30 with papilledema from local database.	Sensitivity 84.1% Specificity 90.6% Accuracy 87.8%
Yang et al. (2019) [48]	To detect optic disc pallor	Classical machine learning	230 images consisting of 107 with disc pallor and 123 normal discs	Sensitivity 95.3% Specificity 96.7% Accuracy 96.1%
Ahn et al. (2019) [49]	To differentiate true ONH swelling from pseudoedema	Deep learning	1396 images (295 with optic neuropathies, 295 with pseudo-papilledema, 779 normal) from local database. Training dataset: 876 Validation dataset: 274 Testing dataset: 219 images	Accuracy 95.9%/96.4%
Milea et al. (2020) [50]	To classify normal optic discs, papilledema, and abnormal optic discs	BONSAI DLS: Segmentation network (Unet) to detect the location of ONH Classification network (DenseNet-121 and DenseNet-201) pretrained on ImageNet	Training dataset: 14,341 images (2148 with papilledema, 3037 with other optic disc abnormalities, 9156 with normal optic discs) from 19 sites and 11 countries Testing dataset: 1505 images (360 with papilledema, 532 with other optic disc abnormalities, 613 with normal optic discs) from 5 other centers in 5 countries.	AUC of 0.99 for discriminating papilledema from normal and abnormal discs and for discriminating normal from abnormal discs 96.4% sensitivity and 84.7% specificity for detecting papilledema
Yang et al. (2020) [51]	To differentiate between NGON and GON	Convolutional neural network (ResNet-50 architecture)	Training dataset: 900 images (300 normal ONH, 300 NGON, 300 GON) Validation dataset: 240 images (80 normal ONH, 80 NGON, 80 GON) Testing dataset: 2675 images (2503 normal ONH, 66 NGON, 106 GON) Images	Accuracy to detect GON: Sensitivity 93.4% specificity, 81.8% AUPRC 0.874 average precision
Biousse et al. (2020) [52]	To distinguish papilledema from normal ONH and ONHs with other abnormalities To distinguish other ONH abnormalities from normal ONH To distinguish normal ONH from papilledema and ONHs with other abnormalities	Deep learning	Training dataset: 14,341 images (2148 with papilledema, 3037 with other optic disc abnormalities, 9156 with normal optic discs) from 19 sites and 11 countries Testing dataset: 800 images (201 with papilledema, 199 with other optic disc abnormalities, 400 with normal optic discs)	Sensitivity (%), Specificity (%), AUC, Accuracy (%) 1. 83.1, 94.3, 0.96, 91.5 2. 73.9, 89.9, 0.89, 89.9 3. 91.0, 93.3, 0.97. 92.1
Vasseneix et al. (2021) [9]	To grade papilledema severity into Mild/Mod (MFS 1–3) vs. Severe (MFS 4 and 5)	Deep learning—Segmentation network (U-net) Classification network (VGGNet) pretrained on ImageNet	Training dataset: 2103 images (1052 with mild/moderate papilledema, 1051 with severe papilledema) from 16 sites and 11 countries Testing dataset: 214 images (92 with mild/moderate papilledema, 122 with severe papilledema) from 4 sites	Sensitivity 91.8%, Specificity 82.6%, AUC 0.93, Accuracy 87.9%
Liu et al. (2021) [53]	To differentiate normal and abnormal ONH To differentiate normal and abnormal ONH using fundus images taken with smartphone and ophthalmic imaging adapter	Deep learning—Classification with ResNet-152 network	Training dataset: 944 images (364 abnormal, 580 normal) from a local database. Testing dataset: 151 images (71 abnormal, 80 normal) from local database, of which 12 images (8 abnormal, 4 normal) were taken using a smartphone.	Sensitivity (%), Specificity (%), AUC, Accuracy (%) 1. 94.0, 96.0., 0.99, - 2. 100.0, 50.0, -, 83
Vali et al. (2023) [54]	To differentiate GON from NGON	Deep learning—DenseNet121	/	Sensitivity 95.36%, specificity 92.19%, accuracy 95.35%

AI, artificial intelligence; AUC, area under the curve; AUPRC, area under the precision recall curve; GON, glaucomatous optic neuropathy; MFS, modified Frisen scale; NGON, nonglaucomatous optic neuropathy; ONH, optic nerve head.

**Table 2:** Comparison of AI model and performance in neuro-ophthalmology studies using OCT.

Authors	Objective	Algoritm	Performance Characteristics
Motamedi et al. 2022 [81]	To distinguish ON from healthy control eyes using DL on peripapillary ring scans	Convolutional neural network	Classification network: AUC of 0.86, accuracy of 0.85 pRNFL: AUC of 0.77
Ciftci Kavaklioglu et al. 2022 [82]	To identify and differentiate structural retinal features in MS, MOGAD, NMOSDs, and monoADS using ML on OCT measures in children	Multiple supervised machine learning classifiers	75% accuracy for DDs 80% accuracy for MS
Kenney et al. 2022 [83]	Machine learning classification of MS from normal controls using SD-OCT and visual measured derived scores	Logistic regression, support vector machine	AUC of 0.89 (95% CI 0.85–0.93) Sensitivity of 81% Specificity of 80%
Bowd et al. 2022 [84]	Comparison of convolutional neural network analysis of vessel density images to GBC analysis of OCT-A vessel density measurements and OCT RNFL thickness measurements for classifying healthy and glaucomatous eyes	Convolutional neural network Gradient Boosting Classifier (GBC)	AUPRC: 0.97 (95% CI 0.95–0.99) GBC AUPRCs $0.87 for vessel density and RNFL thickness measurements
Girard et al. 2023 [85]	To develop a DL algorithm to identify ONH structures using OCT and to differentiate ODD, papilledema, and healthy ONHs	Deep learning segmentation algorithm (Unet++) and classification algorithm (random forest)	Classification: AUC of 0.99 ± 0.001 for ODD detection, 0.99 ± 0.005 for papilledema detection, 0.98 ± 0.01 for detection of healthy ONHs
Li et al. 2024 [86]	To predict disease progression toward papilledema or glaucoma using AI on OCT	Modified convolutional neural network, VGG-19 model	Papilledema: 0.714 precision, 0.769 recall when model trained with RNFL thickness map; AUC of 0.826 Glaucoma: 0.682 precision, 0.857 recall when trained with extracted vertical tomogram; AUC of 0.785
Jalili et al. 2024 [87]	Evaluation of the classification performance of a machine learning algorithm based on vessel density features of OCT-A images for classifying healthy, nonarteritic anterior ischemic optic neuropathy, and optic neuritis	Support Vector Machine (SVM) Random Forest (RF) Gaussian Naive Bayes	All models achieved AUC of 1 and accuracy of 1, at a 50% threshold in discriminating ON from NAION SVM and RF achieved AUC of 1 and accuracy of 1 at 100% threshold in discriminating ON from normal SVM and RF achieved AUC of 1 and accuracy of 1 at 50% threshold in discriminated NAION from normal

AUC, area under the curve; AUPRC, area under the precision recall curve; DD, demyelinating disorders; ML, machine learning, MOGAD, myelin oligodendrocyte glycoprotein antibody-associated disease; monoADS, monophasic acquired demyelinating syndromes; MS, multiple sclerosis; NMOSD, neuromyelitis optical spectrum disorders; OCT, optical coherence tomography; OCT-A, optical coherence tomography angiography; ODD, optic disc drusen; ON, optic neuritis; ONH, optic nerve head; RNFL, retinal nerve fiber layer.

**Table 3:** AI limitations.

Aspect	Limitations
Diagnostic Accuracy	Model overfitting in rare conditions
Workflow Efficiency	Time-intensive validation and tuning
Access and Equity	Limited infrastructure in low-resource areas
Interpretability	Black-box models limit clinical trust
Data Use	Privacy, data ownership concerns
Scalability	Lack of large annotated datasets

**Table 4:** Device based overview for AI implementation.

Tool	What We Know	What We Don’t Know	What We Should Know
OCT	AI models are highly effective at evaluating RNFL and GCC thinning to track axonal damage. DL algorithms help differentiate true optic disc swelling from structural mimics (like buried drusen) Machine learning models are increasingly capable of identifying microscopic retinal biomarkers associated with central nervous system diseases, such as AD, PD and MS. Excellent at evaluating chronic RNFL atrophy and recognizing spatial patterns using CNNs.	The “Why”: Neural networks can flag damage, but they frequently lack interpretability. They cannot explain the specific biological mechanism driving the thinning. Non-Specific Fingerprints: There is “no specific fingerprint” on OCT for complex neurological conditions. For example, AI cannot definitively differentiate between patterns of atrophy caused by glaucoma, optic neuritis, or hereditary neuropathies. Most models perform well at detecting chronic atrophy, but their accuracy is modest when trying to diagnose acute cases of optic neuritis. AI models accurately identify established optic disc swelling, but performance drops significantly when differentiating mild papilledema (indicating increased intracranial pressure) from pseudopapilledema. AI models trained on OCT scans from one manufacturer often generalize poorly when analyzing raw data from another machine. training datasets lack the massive numbers required to train highly reliable, diverse, and robust deep-learning models.	AI should act as a decision-support tool rather than an autonomous diagnostic system.
Fundus Photographs	AI excels at analyzing fundus photographs to distinguish true optic disc swelling (often a sign of life-threatening increased intracranial pressure) from benign, congenital disc anomalies. Specialized algorithms (like those from the BONSAI Consortium) are achieving high diagnostic accuracy (often >93%) when differentiating acute optic neuritis from other ischemic optic neuropathies. AI models can detect subtle changes in retinal blood vessel geometry and nerve fiber layers to identify early signatures of neurodegenerative diseases, including AD and PD, long before clinical symptoms present. In environments without immediate access to subspecialists, AI algorithms flag high-risk images for urgent triage and referral. AI systems can automatically filter and select high-quality, readable fundus photographs from large datasets	Most AI models are trained retrospectively on narrow or specific demographic datasets, which can limit their generalizability across different ethnicities. AI algorithms have difficulties in identifying acute optic neuritis or separate mild true swelling from pseudopapilledema. AI often fails when attempting to perform complex differential diagnoses—it cannot consistently distinguish between different patterns of chronic optic atrophy Algorithms are typically fine-tuned for specific high-end devices. We don’t fully know how they scale when evaluating images from varied, real-world community settings. It is unknown how these AI models perform across different retinal pigmentations and rare anatomical variations Many deep learning models rely on heatmaps (like Grad-CAM) to show where they are looking, but we do not fully understand the exact high-dimensional features the AI uses to make definitive calls	prospective validation of AI analyzing fundus photos to predict brain decline standardized, widely adopted AI frameworks that seamlessly fuse fundus photography with Optical Coherence Tomography (OCT) and clinical metadata AI algorithms are designed to serve as diagnostic decision support tools, not a substitute for a comprehensive clinical evaluation by an ophthalmologist.
Radiomics	AI radiomics applied to brain MRIs is used to predict the etiology of optic neuritis and accurately localize lesions along the visual pathway. When assessing tumors pressing on the optic chiasm (such as pituitary adenomas), MRI radiomic signatures are being used to predict tumor invasiveness, consistency, and surgical risk	Clinicians still lack a clear understanding of the exact, granular pixel-level features these algorithms use to make determinations. We do not yet know how to perfectly fuse retinal radiomics (OCT, fundus photos) with neurological imaging (MRI) and clinical data (symptom timelines, visual field tests) in a cohesive, real-time diagnostic pipeline. Studies are frequently retrospective and rely on single-center datasets. It is largely unknown how models will perform when exposed to unrepresented demographics, different camera hardware, or rare disease phenotypes, leading to potential dataset bias. The exact legal and ethical frameworks remain unresolved.	The latest systems are moving toward multimodal data, combining structural images (MRI/OCT), behavioral data (visual fields), and clinical biomarkers to provide a comprehensive diagnostic picture.

## Data Availability

The original contributions presented in this study are included in the article. Further inquiries can be directed to the corresponding author.

## Abbreviations

The following abbreviations are used in this manuscript:

AD	Alzheimer Disease
ADS	Acquired Demyelinating Syndrome
AI	Artificial Intelligence
AUC	Area under the receiver operating characteristic curve
AUPRC	Area under the precision recall curve
BCI	Brain-computer interface
CNN	Convolutional neural network
CNS	Central nervous system
DD	Demyelinating disease
DL	Deep learning
DLS	Deep learning segmentation
FAZ	Foveal avascular zone
GC-IPL	Ganglion cell Inner Plexiform layer
GCL	Ganglion cell layer
GON	Glaucomatous optic neuropathy
LLM	Large Language Models
MFS	Modified Frisen scale
ML	Machine learning
MLLMs	Multimodal Large Language Models
MOG	Myelin oligodendrocyte glycoprotein
MOGAD	Myelin oligodendrocyte glycoprotein antibody-associated disease
MRI	Magnetic resonance imaging
MS	Multiple sclerosis
NAION	Non-arteritic anterior ischemic optic neuropathy
NGON	Non-Glaucomatous optic neuropathy
NMOSD	Neuromyelitis optica spectrum disorder
OCT	Optical coherence tomography
OCTA	Optical coherence tomography angiography
ODP	Optic disc pit
ON	Optic Neuritis
ONH	Optic Nerve Head
PD	Parkinson Disease
QSM	Quantitative Susceptibility Mapping
RNFL	Retinal nerve fiber layer
SD-OCT	Spectral Domain Optical Coherence Tomography
VEP	Visual evoked potentials
XAI	Explainable AI
